# Supplementary material for: Modulated Electro-Hyperthermia Accelerates Tumor Delivery and Improves Anticancer Activity of Doxorubicin Encapsulated in Lyso-Thermosensitive Liposomes in 4T1-Tumor-Bearing Mice
Source: Int J Mol Sci. 2024 Mar 7;25(6):3101. doi: 10.3390/ijms25063101 (PMC10970314; doi:10.3390/ijms25063101)
Supplement: Supplementary file 1 [file ijms-25-03101-s001.zip › ijms-2850047-supplementary.pdf]

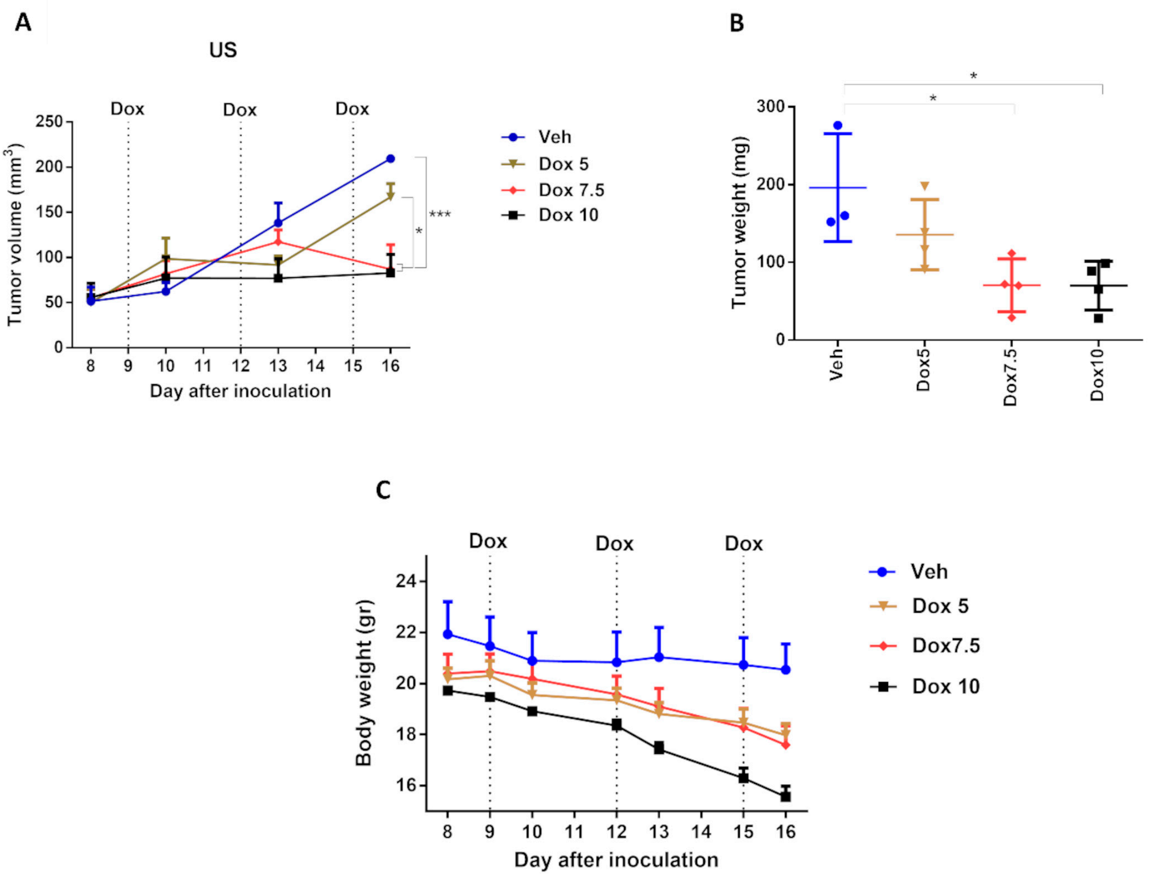

Figure S1. Effective and tolerated doses of DOX in 4T1-bearing mice after three treatments. Tumor volume by Ultrasound (US) (**A**). Tumor weight (**B**). Body weight change (**C**). Mean ± SEM, (**A**, **C**) two-way ANOVA, (**B**) one- way ANOVA *: $p < 0.05$, ***: $p < 0.001$.

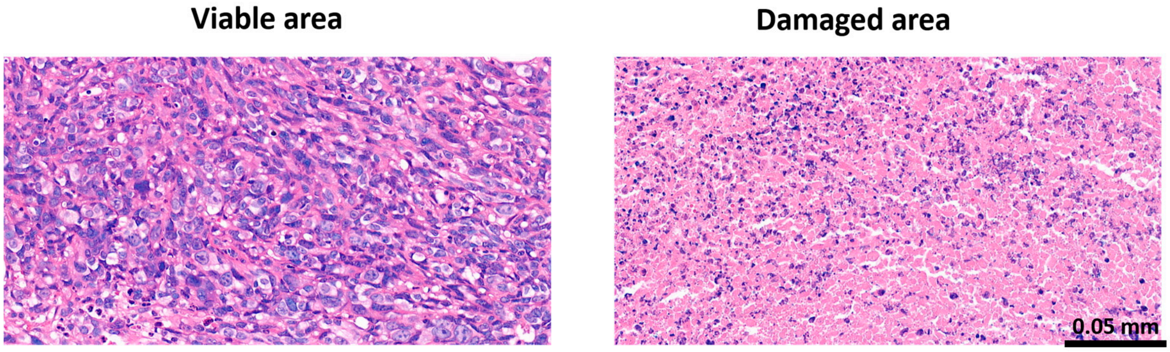

Figure S2. The difference between the viable and damaged areas on hematoxylin-eosin-stained sections.
